# Implication of IL-7 receptor alpha chain expression by CD8^+^ T cells and its signature in defining biomarkers in aging

**DOI:** 10.1186/s12979-022-00324-6

**Published:** 2022-12-21

**Authors:** Min Sun Shin, Hong-Jai Park, Juan Young, Insoo Kang

**Affiliations:** 1 Departments of Internal Medicine, Section of Rheumatology, Allergy & Immunology, Yale University School of Medicine, S525C TAC, 300 Cedar Street, New Haven, CT 06520 USA; 2 Departments of Psychiatry, Yale University School of Medicine, New Haven, CT 06520 USA

**Keywords:** Aging, CD8^+^ T cells, IL-7 receptor alpha chain, Signature, Biomarkers

## Abstract

CD8^+^ T cells play an important role in host defense against infections and malignancies as well as contribute to the development of inflammatory disorders. Alterations in the frequency of naïve and memory CD8^+^ T cells are one of the most significant changes in the immune system with age. As the world population rapidly ages, a better understanding of aging immune function or immunosenescence could become a basis for discovering treatments of illnesses that commonly occur in older adults. In particular, biomarkers for immune aging could be utilized to identify individuals at high risk of developing age-associated conditions and help monitor the efficacy of therapeutic interventions targeting such conditions. This review details the possible role of CD8^+^ T cell subsets expressing different levels of the cytokine receptor IL-7 receptor alpha chain (IL-7Rα) and the gene signature associated with IL-7Rα as potential biomarkers for immune aging given the association of CD8^+^ T cells in host defense, inflammation, and immunosenescence.

## Introduction

Aging is a natural process that arises inevitably in every living organism and affects all organ systems of the human body. The immune system which comprises cells, tissues and organs provides host defense against infections and other diseases through a complex network of biological processes. Alterations in the immune system occur with age, likely contributing to the development of many pathologic conditions such as malignancy, infections and inflammatory diseases frequently found in older adults [1–4]. As the world’s population shifts to an increasingly older demographic, it is critical to understand how aging affects the immune system and whether such changes could be delayed, inhibited, or reversed allowing for the prevention or treatment of age-associated pathologic conditions. In addressing these questions, biomarkers for immune aging or immunosenescence could serve as essential tools which may identify individuals at high risk for the development of age-associated conditions and allow for more accurate evaluations of the therapeutic efficacy of pharmacologic interventions.

Alterations in the frequency of naïve and memory CD8^+^ T cells are one of the most significant changes in immunosenescence. With age, the frequency of naïve CD8^+^ T cell decreases while the frequency of memory CD8^+^ T cells increases. We demonstrated an expansion of effector memory (EM) CD8^+^ T cells expressing low levels of T cell homeostasis cytokine IL-7 receptor alpha chain (IL-7Rα or CD127) in older adults [5, 6] as well as the possible contribution of such change to an age-associated gene signature in peripheral blood [7]. An independent investigation also reported the silencing of the *IL7R* gene and the IL-7 signaling pathway genes in human memory CD8^+^ T cells [8]. A study of a nonagenarian population and its middle-aged controls found high levels of *IL7R* gene expression in peripheral blood with reduced mortality [9], raising the biological significance of altered expression of *IL7R* and related genes, especially in memory CD8^+^ T cells. This manuscript will review the possible value of CD8^+^ T cell subsets expressing distinct levels of IL-7Rα and gene signature associated with this cytokine receptor as potential biomarkers for immune aging in humans.

### Overview of CD8^+^ T cell homeostasis and function

The development of CD8^+^ T cells occurs in the thymus where the progenitor cells of T cells from the bone marrow undergo a complicated selection process to produce cells with the optimal affinity for major histocompatibility complex (MHC) molecules while circumventing self-antigen reactive cells [10]. Immature CD8^+^ T cells or thymocytes which have survived these selection processes (referred to positive and negative selections) are exported into circulation as naïve but mature CD8^+^ T cells. Expressing high levels of lymphoid tissue homing molecules like chemokine receptor (CCR) 7, naïve T cells migrate to the secondary lymphoid tissues where they inspect antigens presented by antigen presenting cells (APCs) such as dendritic cells (DCs) [11–13]. The naïve CD8^+^ T cells which have recognized the appropriate antigen with the T cell receptor (signal 1) become activated, proliferate, and differentiated into effector T cells, with the help from cognate interactions through co-stimulatory molecules and cytokines (signals 2 and 3, respectively). The effector CD8^+^ T cells now have potently express cytotoxic molecules and cytokines such as perforin, granzymes, IFN-γ, and TNF-α through upregulating the transcription factors T-bet and eomesodermin [14, 15]. The effector CD8^+^ T cells also express chemokine receptors with the capacity to dictate their migration to the sites of infection and/or inflammation where the secreted IFN-γ and TNF-α from the migrated effector CD8^+^ T cells can activate additional immune cells (e.g.*,*, monocytes, natural killer (NK) cells, T cells) and non-immune tissues like endothelial cells [16, 17]. When the source of immunogens such as microbial antigens is cleared, most effector CD8^+^ T cells undergo activation-induced cell death. However, a small number of effector T cells survive and become memory CD8^+^ T cells providing long-term immune protection against the same pathogens [1]. The memory CD8^+^ T cells can rapidly turn into effector cells, even in the absence of co-stimulatory processes and cytokines, when they reencounter the antigen. Memory CD8^+^ T cells continue to divide at a slow rate in the absence of antigen; with cytokines IL-7 and IL-15 promoting T cell maintenance [18–20]. IL-7 is largely produced by thymic epithelial and bone marrow stromal cells while the major source of IL-15 includes cells of myeloid origin like monocytes, macrophages and DCs [20–22].

### Heterogeneity exists in memory CD8^+^ T cells

**Fig. 1:**
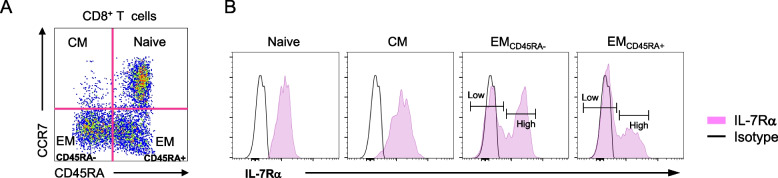
CD8^+^ T cell subsets with distinct characteristics, including IL-7 receptor alpha expression, can be identified in human peripheral blood. Flow cytometric analysis of human peripheral CD8^+^ T cells showing (**a**) naïve (N), central (CM), CD45RA^−^ and CD45RA^+^ effector memory (EM) CD8^+^ T cells (**b**) expressing distinct levels of IL-7 receptor alpha chain

Memory CD8^+^ T cells are heterogeneous populations. Based on their capacity to migrate to secondary lymphoid tissue (e.g.*,* spleen, lymph nodes) and infected or inflamed peripheral sites, memory CD8^+^ T cells can be categorized into central (CM) and effector memory (EM) CD8^+^ T cells [1]. CM CD8^+^ T cells that express lymphoid tissue homing chemokine receptor 7 (CCR7) migrate to secondary lymphoid tissues where the chemokines CCL19 and 21, ligands for CCR7, are highly expressed. In contrast, EM CD8^+^ T cells can travel to peripheral tissues through the expression of the receptors for the molecules present in inflamed tissues, but not CCR7. Using flow cytometry, naïve, CM, CD45RA^−^ EM CD8^+^ and CD45RA^+^ EM (TEMRA) CD8^+^ T cells can be identified based on the expression of CCR7 and CD45RA, T cell receptor co-receptor (Fig. 1A) [5]. Human CD45RA^−^ and CD45RA^+^ EM CD8^+^ T cells have two different cell subsets expressing high and low levels of IL-7Rα, the high affinity receptor chain for T cell homeostatic cytokine IL-7 (Fig. 1B) [5]. IL-7Rα^high^ and ^low^ EM (referred to both CD45RA+/− cells unless specified) CD8^+^ T cells have distinct traits as discussed below (also see Table 1).

**Table 1:** Human IL-7 receptor alpha (IL-7Rα) high and low effector memory* CD8^+^ T cells have distinct cellular characteristics

	IL-7Rα^high^ cells	IL-7Rα^low^ cells
CD27 and CD28 expression	High	Low
CD57 expression	Low	High
Perforin, granzyme B	Low	High
T cell receptor (TCR) repertoire	Diverse	Limited
TCR-mediated proliferation	High	Low
IFN-γ and TNF-α production	Low	High
IL-7 survival response	High	Low
IL-15-mediated proliferation	Low	High
*IL7R* gene expression	High	Low
DNA Methylation in *IL7R* promoter	Low	High
CX3CR1 (fractalkine receptor) expression	Low	High
DNA Methylation in *CX3CR1* promoter	High	Low
Aging	Low	High

*effector memory CD8^+^ T cells are CCR7^−^ and CD45RA^+/−^

### Age-associated changes in CD8^+^ T cell subsets

Alterations in CD8^+^ T cell immunity occur with age. These include impaired cellular functions like cytotoxicity as well as changes in the subsets of naïve and memory CD8^+^ T cells. With aging, the frequency of naïve CD8^+^ T cell decreases while the frequency of memory CD8^+^ T cells, including both CD45RA^−^ and ^+^ EM (TEMRA) CD8^+^ T cells, increases [23–25]. In older adults, oligoclonally expanded populations of memory CD8^+^ T cells are found [23, 24, 26], suggesting that such memory cell expansion could be driven in part by repeated exposures to microbial and viral antigens over a lifetime. Indeed, the expanded memory CD8^+^ T cells constitute CD28^−^ (T cell surface molecule downregulated in antigen-experienced cells) and CD57^+^ (replication senescence marker) EM CD8^+^ T cells, which also include terminally differentiated TEMRA cells [27, 28]. Also, the infectious status of cytomegalovirus (CMV), a virus known to establish life-long latent infection, has been associated with expansion of memory CD8^+^ T cells including oligoclonally expanded cells in older adults [29–32]. Although such a relationship supports the implication of chronic antigenic stimulation (e.g., through CMV infection) in expanding EM CD8^+^ T cells with aging, CD8^+^ T cell clonal expansion was found in aged mice lacking a MHC class I molecule as well as in aged mice repeatedly injected with adjuvant alone [33]. These findings suggest the implication of an antigen-independent mechanism in expanding memory CD8^+^ T cells in older adults, through potential pathways involving IL-15- and/or IL-7-mediated CD8^+^ T cell maintenance.

Older adults have expansion of IL-7Rα^low^ effector memory (EM) CD8^+^ T cells that express high levels of inflammatory molecules in association with distinct global DNA methylation and gene expression profiles.

While investigating the mechanisms and significance of memory CD8^+^ T cell expansion with age in humans, we measured the expression of IL-7Rα (CD127) on human CD8^+^ T cells in young (age ≤ 40) and older (age ≥ 65) adults, considering the possible role of this cytokine receptor with the capacity to promote memory T cell survival. We found that human naïve and CM CD8^+^ T cells expressed high levels of IL-7Rα while human CD45RA^−^ and CD45RA^+^ EM CD8^+^ T cells had two cell subsets expressing high and low levels of IL-7Rα with distinct cellular characteristics (Fig. 1B and Table 1) [5]. For example, IL-7Rα^low^ EM CD8^+^ T cells exhibit unique DNA methylation and gene expression profiles as compared to IL-7Rα^high^ EM CD8^+^ T cells [5–7, 17, 34–36] (see Table 1). In addition, IL-7Rα^low^ EM CD8^+^ T cells demonstrate greater cytotoxic properties as reflected by higher expression of perforin and/or granzyme B compared to IL-7Rα^high^ EM CD8^+^ T cells. IL-7Rα^low^ EM CD8^+^ T cells also produce much higher levels of IFN-γ and TNF-α and were found to have increased expression of inflammatory chemokine receptors such as CX3CR1 which binds its ligand CX3CL1 (fractalkine), a chemokine involved in inflammatory conditions like atherosclerosis [17, 37]. IL-7Rα^low^ EM CD8^+^ T cells also exhibited increased chemotaxis in response to CX3CL1 and potently induced CX3CL1 expression on human endothelial cells via secreting IFN-γ and TNF-α, suggesting this cell population’s possible role in vascular inflammation [17]. DNA hypo- and hyper-methylation are associated with active and inactive gene expression, respectively [38]. Of note, IL-7Rα^low^ and IL-7Rα^high^ EM CD8^+^ T cells have distinct genome-wide DNA methylation profiles, suggesting the role of DNA methylation in conferring unique cellular characteristics in these cell subsets. Representative of this, IL-7Rα^low^ EM CD8^+^ T cells were found to have increased DNA methylation in the *IL7RA* gene promoter while exhibiting decreased DNA methylation in the *CX3CR1* gene promoter [17, 35]. As it is known that aging can affect DNA methylation [39, 40], these findings raise a possible role for DNA methylation in defining the characteristics of CD8^+^ T cell subsets including naïve, IL-7Rα^low^ and ^high^ EM CD8^+^ T cells and altering the gene expression profile of these cell subsets with age.

Although we initially considered the possible implication of IL-7 in expanding memory CD8^+^ T cells with age, the results of our study demonstrated the expansion of IL-7Rα^low^ EM CD8^+^ T cells in older adults compared to young adults [5], indicating that the expansion of EM CD8^+^ T cells with age was not secondary to IL-7. The expansion of IL-7Rα^low^ EM CD8^+^ T cells was more prominent in older adults infected with CMV [41], suggesting a role of chronic and/or repetitive antigenic stimulation in inducing this phenomenon. Indeed, IL-7Rα^low^ EM CD8^+^ T cells are largely antigen-experienced (CD27^−^CD28^−^) cells [5]. The soluble form of IL-7Rα without the transmembrane domain can be generated by alternative splicing [42] although the membrane bound form of IL-7Rα also may also be cleaved [43]. Low levels of the membrane bound IL-7Rα on IL-7Rα^low^ EM CD8^+^ T cells could be from increased production of alternatively spliced soluble IL-7Rα. However, our published study demonstrated that decreased mRNA expression of the *IL7RA* gene in IL-7Rα^low^ EM CD8^+^ T cells was secondary to increased DNA methylation in the *IL7RA* promoter region and reduced promoter activity [35]. Still, the effect of aging on soluble IL-7Rα expression is unknown and warrants further investigation. In our recent study, we explored the effect of aging on the multi-dimensional characteristics and heterogeneity of CD8^+^ T cells in peripheral blood of young and older adults using mass cytometry or Cytometry by Time-Of-Flight (CyTOF) with computational algorithms. A subset of CD8^+^ T cells with the characteristics of IL-7Rα^low^ TEMRA CD8^+^ T cells expanded in older adults, and such cells had unique characteristics of expressing high levels of the chemokine receptors, including CXCR1, CXCR5, CXCR6, and CX3CR1, the co-stimulatory molecule 4-1BB, and CD57 with low expression levels of CD27 and CD28 [6]. Although IL-7Rα^low^ EM CD8^+^ T cells had impaired T cell receptor (TCR)-mediated proliferation, they proliferated in response to IL-15 [44]. In fact, IL-7Rα^low^ but not ^high^ EM CD8^+^ T cells from older adults had increased proliferative response to IL-15 compared to those from young adults (Shin and Kang, unpublished data). Also, older adults were found to have greater IL-15 production from monocytes compared to young adults [45]. These findings suggest the role of chronic repetitive antigenic stimulation like CMV and the cytokine IL-15 in expanding IL-7Rα^low^ EM CD8^+^ T cells with aging.

Human IL-7Rα^low^ EM CD8^+^ T cells have a set of genes with altered expression that corresponds to age-associated genes in peripheral blood.

A meta-analysis on the global gene expression profile of human peripheral whole blood from ~ 15,000 individuals identified 1497 genes that were associated with chronological age [46]. Such genes were those related to T and B cell signaling, innate immunity, and hematopoiesis, likely reflecting the presence of different types of circulating blood cells that could change with age [46]. Of note, about one third (231/774) of the differentially expressed genes in IL-7Rα^low^ EM CD8^+^ T cells vs. IL-7Rα^high^ EM CD8^+^ T cells corresponded to 15% (231/1497) of the age-associated genes identified by the meta-analysis [7]. The expression fold changes of these genes between IL-7Rα^low^ and ^high^ EM CD8^+^ T cells and age z-scores of the genes from the meta-analysis were highly correlated [7]. Among these genes (i.e., aging signature genes) of IL-7Rα^low^ EM CD8^+^ T cells, the genes encoding the cytotoxic molecules GZMH, GZMB, FGFBP2, and the chemokine receptor CX3CR1 were the ones with the highest levels of age-associated z scores and expression fold change between IL-7Rα^low^ and ^high^ EM CD8^+^ T cells [7]. Our transcriptional regulatory network analysis revealed that the age-associated signature genes of IL-7Rα^low^ EM CD8^+^ T cells could be regulated by a set of transcriptional factors encoded by *MYC*, *BATF, SATB1*, *KLF4, IRF1*, and *NFKB1* [7]. We selected the genes with the 10 highest aging z-scores which were upregulated at 2-fold or greater levels in IL-7Rα^low^ EM CD8^+^ T cells. The expression levels of these genes, which included *GZMH, FGFBP2, SYT11, NUAK1, TGFBR3, NKG7, GZMB, CX3CR1, PRSS23,* and *OSBPL5*, had an association with influenza vaccine responses in older adults. The top 10 gene scores calculated based on the expression levels of these top 10 genes were significantly higher in influenza vaccine responders than in non-responders in older adults [7], suggesting the possible utility of aging signature genes of IL-7Rα^low^ EM CD8^+^ T cells in predicting influenza vaccine response in older adults. The possible mechanism for this finding could be related to the inflammatory characteristics of IL-7Rα^low^ EM CD8^+^ T cells which express high levels of chemokine receptors (e.g.*,* CX3CR1, CXCR1), inflammatory cytokines (IFN-γ, TNF-α), and cytotoxic molecules (e.g.*,* perforin, granzyme B) [7, 17]. With these chemokine receptors, IL-7Rα^low^ EM CD8^+^ T cells can move to a vaccine-injected site and secrete IFN-γ and TNF-α which may enhance the local immune response to the vaccinated antigen like an adjuvant [7].

### *IL7R* and its related genes as biomarkers of aging

Expression of the *IL7R* gene and its related molecules in peripheral blood was associated with longevity based on the Leiden Longevity Study in Netherlands which comprised nonagenarian sibling pairs, their middle-aged offspring, and the partners of the offspring as population controls [9, 47, 48]. The original clinical report of the Leiden Longevity Study found a lower mortality rate of nonagenarian siblings compared to sporadic nonagenarians over an average of 2.7 and 3 years of follow-up, respectively [48]. The offspring of nonagenarian siblings had a lower prevalence of morbidity, including myocardial infarction and diabetes mellitus, and use of cardiovascular medicines than their partners. These findings raise the notion that resilience against disease and death have similar underlying biological mechanisms that are determined by genetic or familial factors [48]. Using peripheral blood samples from the Leiden Longevity Study, a transcriptomic analysis of whole blood by gene expression microarray and follow-up RT-qPCR analysis identified that the *IL7R* gene was one of the 21 genes identified as an aging gene signature. In this study, the *IL7R* gene was significantly decreased in peripheral blood of the middle-aged offspring of the nonagenarians compared to the controls, suggesting an association of the *IL7R* expression with longevity in middle age [47]. However, this notion has been challenged by the results of a separate study from the same research group showing that high levels of *IL7R* gene expression was associated with reduced mortality over 10 years in nonagenarians and middle-aged controls [9]. The authors also investigated 6 IL-7Rα-interacting genes, including *IL2RG*, *IL7*, *TSLP*, *CRLF2*, *JAK1* and *JAK3*, which were analyzed in peripheral whole blood by RT-qPCR [9]. These genes are directly involved in IL-7R signaling. The *IL2RG* gene encodes IL-2 receptor subunit gamma or common cytokine receptor gamma chain (CD132 or γC) which forms the IL-7R complex with the high affinity IL-7Rα chain [20]. IL-7 binding to the IL-7R complex induces the activation of JAK1, JAK3, and STAT5, leading to upregulation of anti-apoptotic molecule Bcl-2 [20]. The *CRLF2* gene encodes cytokine receptor like factor 2 (CRLF2) which forms the receptor for thymic stromal lymphopoietin (TSLP) together with IL-7Rα [49]. TSLP, which is mainly produced by non-hematopoietic cells including thymic stromal cells, epithelial cells and fibroblasts, can affect the function of multiple immune cells including dendritic cells and T cells [49]. Decreased levels of circulating IL-7 were reported in humans with age, which could be related to age-associated thymic atrophy [50, 51] although the effect of aging on circulatory levels of TSLP is unknown. Of interest, the expression levels of *IL7R, IL2RG, IL7, TSLP*, and *CRLF*2 genes in peripheral blood were lower in nonagenarians than in middle-aged controls [9], suggesting an age-associated alteration in the expression of IL-7R and its related genes in peripheral blood. However, the biological significance of such changes has yet to be elucidated.

The possible significance of the *IL7R* gene and the IL-7 signaling pathway genes as biomarkers of aging was suggested by the results of a study which analyzed chromatin accessibility and transcriptomics of peripheral blood mononuclear cells (PBMCs) in young and older adults using systems immunology approaches [8]. In this study, the authors analyzed chromatin accessibility in total PBMCs and purified monocytes, B and T cells from healthy young and older adults using the assay for transposase-accessible chromatin with sequencing (ATAC-seq). The authors found that the *IL7R* gene was among the top genes linked to multiple closing peaks (i.e., loss of chromatin accessibility) accompanied by aging-associated decreases in *IL7R* gene expression [8]. Additional genes in the IL-7 signaling cascade, including *JAK1*, *JAK3*, *STAT5A*, and *STAT5B*, also had decreased chromatin accessibility in older individuals, possibly accounting in part for the impaired signaling and responses to IL-7 in CD8^+^ T cells of older adults [5, 8]. This signature was likely from memory CD8^+^ T cells since chromatin inaccessibility at the *IL7R* promoter was specific for memory CD8^+^ T cells and older adults had decreased expression of IL-7R gene and protein [8]; a finding substantiated by our previous study that demonstrated an increase in the frequency of IL-7Rα^low^ EM CD8^+^ T cells in older adults [5]. In addition, there was no or minimal changes in chromatin accessibility in CD4^+^ T cells, monocytes, and naïve B cells with age [8]. In contrast to the *IL7R* gene, genes encoding cytotoxic molecules such as granzyme H and granulysin had open chromatin in PBMCs of older adults which is reflective of the increased expression of *granzyme B* and *granulyin* in IL-7Rα^low^ EM CD8^+^ T cells that expand with aging [7, 8]. The decreased chromatin accessibility in the *IL7R* gene promoter with decreased IL-7R gene and protein expression in PBMCs of older adults could be related to increased DNA methylation of the *IL7R* gene promoter since we previously reported increased DNA methylation in the *IL7R* gene promoter with decreased IL-7R gene and protein expression by CD8^+^ T cells [35] and DNA methylation affects chromatin accessibility [52]. Overall, these findings support the concept of utilizing altered expression levels of *IL7R* and its associated molecules as biomarkers of an aging immune system in humans.

## Conclusion

**Fig. 2:**
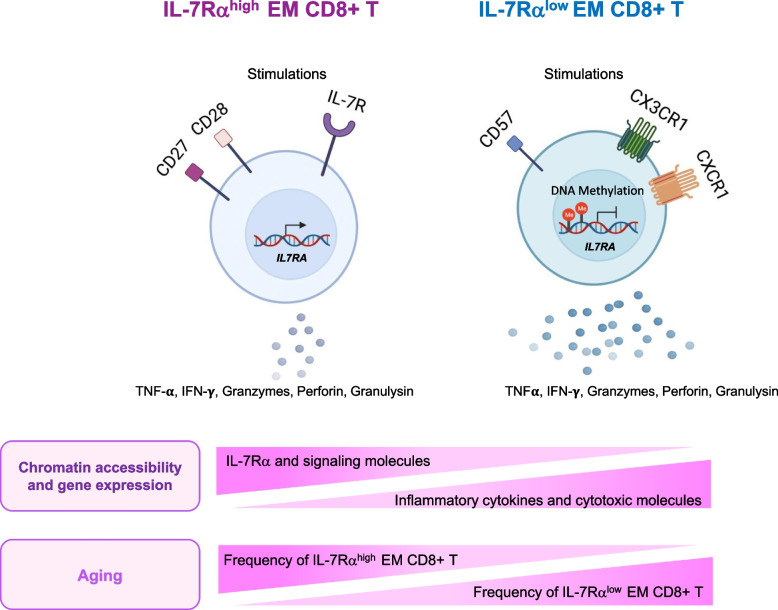
A model for the possible implication of IL-7 receptor alpha (IL-7Rα) and effector memory (EM) CD8^+^ T cells expressing distinct levels of IL-7Rα as biomarkers of aging. IL-7Rα^high^ and ^low^ EM (CCR7^−^) CD8^+^ T cell subsets with distinct characteristics, including the expression of CD27, CD28, CD57, CX3CR1 and CXCR1 as well as the production of inflammatory and cytotoxic molecules (e.g.*,* TNF-α, IFN-γ, granzymes, perforin, and granulysin), are present in human peripheral blood (see Table 1 for summary of cellular characteristics). The differential expression of IL-7Rα is regulated by DNA methylation and chromatin accessibility in the *IL7RA* gene promoter. Alterations in DNA methylation and chromatin accessibility in CD8^+^ T cells occur with aging, contributing to altered expression of IL7RA signaling molecules, inflammatory cytokines, and cytotoxic molecules. The frequency of IL-7Rα^low^ EM CD8^+^ T cells in peripheral blood increases with aging while the frequency of IL-7Rα^high^ EM CD8^+^ T cells decreases

The studies reviewed support the consideration of IL-7Rα and its related molecules, especially in association with memory CD8^+^ T cells, as potential biomarkers for aging in humans (Fig. 2). EM CD8^+^ T cells expressing low levels of IL-7Rα expand in peripheral blood of older adults [5, 6]. Having unique cellar characteristics, including a distinctive gene expression profile, this cell expansion likely contributes to an age-associated gene signature found in peripheral blood of humans with age (Fig. 2) [7]. The relationship of IL-7Rα^low^ EM CD8^+^ T cells with aging is further supported by the results of a study reporting the silencing of the *IL7R* gene and the IL-7 signaling pathway genes in memory CD8^+^ T cells as potential biomarkers of aging [8] as well as increased chromatin accessibility and expression of genes encoding cytotoxic molecules highly expressed by IL-7Rα^low^ EM CD8^+^ T cells [5, 7, 8]. A study of a nonagenarian population and its middle-aged controls found high levels of *IL7R* gene expression in the peripheral blood of nonagenarians with reduced mortality over a span of 10 years [9], raising the possible biological significance of altered expression of *IL7R* and related genes, especially in memory CD8^+^ T cells. Taken together, measuring *IL7R* gene expression levels, the expression of *IL7R* gene associated signaling and network genes, and quantifying IL-7Rα^low^ EM CD8^+^ T cell population frequencies (and their associated gene signature levels) have the potential to serve as biomarkers of aging, warranting additional population based longitudinal studies.

## Data Availability

not applicable.
